# Preliminary results of cannulated screw fixation for isolated pubic ramus fractures

**DOI:** 10.1007/s11751-012-0134-7

**Published:** 2012-05-01

**Authors:** Jasper Winkelhagen, Michel P. J. van den Bekerom, Hugo W. Bolhuis, Mike Hogervorst

**Affiliations:** Department of General Surgery and Traumatology, Gelre Hospitals, Location Lukas, P.O. Box 9014, 7300 DS Apeldoorn, The Netherlands

**Keywords:** Pubic ramus, Fracture fixation, Osteosynthesis, Canulated intramedullary screw, Elderly

## Abstract

Isolated pubic ramus fractures are common fractures in the elderly, and treatment is typically non-operative. Up to 35 % of patients have a prolonged hospital stay due to pain. A small number of these patients do not respond to standard (non-operative) treatment. We retrospectively reviewed six patients with isolated pubic ramus fractures and persistent pain who were treated with percutaneous retrograde pubic ramus screw fixation. The study group consisted of six women with an average age of 81 years (72–86 years). Patients with symptomatic posterior pelvic ring injuries were excluded. All patients showed improvement after surgery, with three patients pain free and three patients with reduced pain. The mean time spent in the hospital was 9 days (range 3–18 days). There were complications post-operatively: two patients had pneumonia, two with confusional states, and one patient had a urinary tract infection. Despite these events, which are associated with surgery in patients with comorbidites from advanced age, retrograde pubic ramus screw fixation is an effective treatment option for patients with persistent pain from isolated pubic fractures.

## Introduction

A pubic ramus fracture after low-energy trauma is a common fracture in the geriatric population. The overall incidence of pubic ramus fractures in the general population is 6.9/100,000/year, with an incidence of 25.6/100,000/year in patients aged over 60 years. [6] Given the high and rising incidence, the impact on healthcare resources is considerable. [7].

The natural course is innocuous in most of the cases. Patients with pubic ramus fractures have a good prognosis with regard to long-term pain relief and functional outcome [8], but hospital admission is often needed and mobilization can be painful and time-consuming despite analgesics and physical therapy. Many patients do not reach their pre-injury level of mobility, and there is high mortality in these patients [6, 8]. There is need for improvement in the management of pubic ramus fractures in the elderly population, in particular for those patients who do not respond to non-operative treatment. Additionally, a fracture of the pubic ramus may lead to a (symptomatic) non-union rarely. Both these factors call for an operative intervention with a low complication rate. Some case reports have indicated that treatment with a retrograde pubic screw can be a successful option [2, 9]. We postulate that early retrograde screw fixation is a treatment option for patients with pubic ramus fractures who have persisting anterior pain and who do not respond to non-operative management.

## Patients and methods

### Patients

Between January 2002 and the end of December 2006, 297 patients (aged over 60 years) with pubic ramus fractures attended the emergency department of our hospital. Forty needed admission because of severe pain and an inability to return to their residence. The other 257 patients were discharged either to their residence with adequate analgesics and support of their family or directly for temporary admission in a nursing home. Of the forty patients admitted, thirty-two could be mobilized within a few days with analgesics (paracetamol and NSAIDs), walking aids and the supervision of a physiotherapist. This approach was unsuccessful in eight patients who were then treated with percutaneous cannulated screw fixation. Surgery was planned if no progression in weightbearing could be made with non-operative treatment after 3 days. We retrospectively reviewed these patients. Two patients were excluded as they did not represent the sample inclusion criteria accurately: one patient had a non-union of a pubic fracture and was treated successfully with percutaneous cannulated screw fixation more than 1 year after trauma; the second patient had an associated iliac wing fracture for which osteosynthesis was performed. This was considered a more extensive pelvic injury and therefore was excluded. The final sample included 6 women, with an average age of 81 years (range, 72–92 years). Four patients had unilateral and two patients had bilateral superior pubic ramus fractures. All fractures were caused by low-energy trauma. None of the patients had physical symptoms of a posterior pelvic ring injury. The analysed variables included the demographical characteristics, hospital stay, complications, pain at discharge and at follow-up, mobility at discharge and at follow-up, and whether patients were discharged to their original residence. CT scanning was not routinely required in the diagnosis of isolated fractures of the pubic ramus because the low probability of finding associated injuries after such low-energy trauma. Even if associated posterior ring injuries were present, these did not require operative treatment [12].

### Surgical technique

**Fig. 1:**
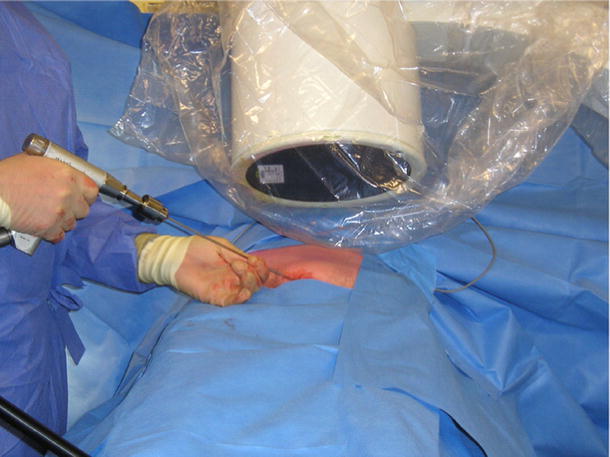
K-wire is inserted in a retrograde direction within the distal medial fragment of the superior ramus pubis

**Fig. 2:**
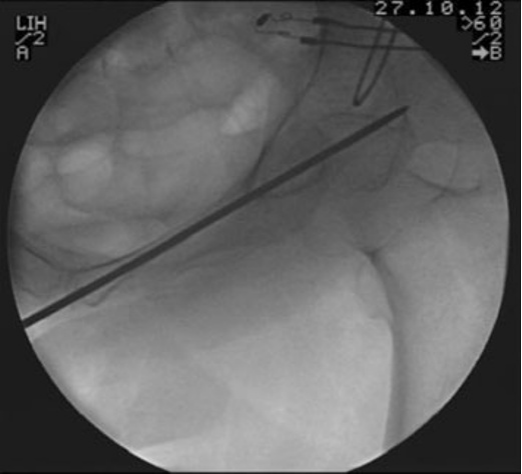
Inlet view: intra-operative guiding K-wire

**Fig. 3:**
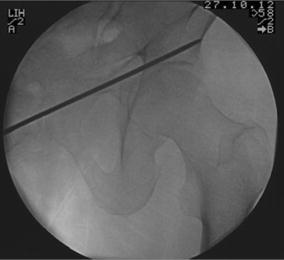
Outlet view: intra-operative guiding K-wire

**Fig. 4:**
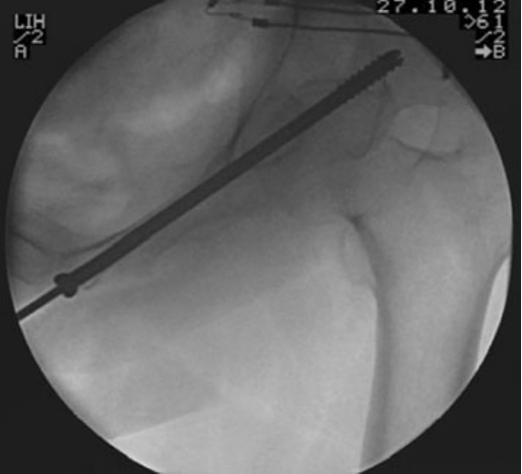
Inlet view: intra-operative canulated screw placement

**Fig. 5:**
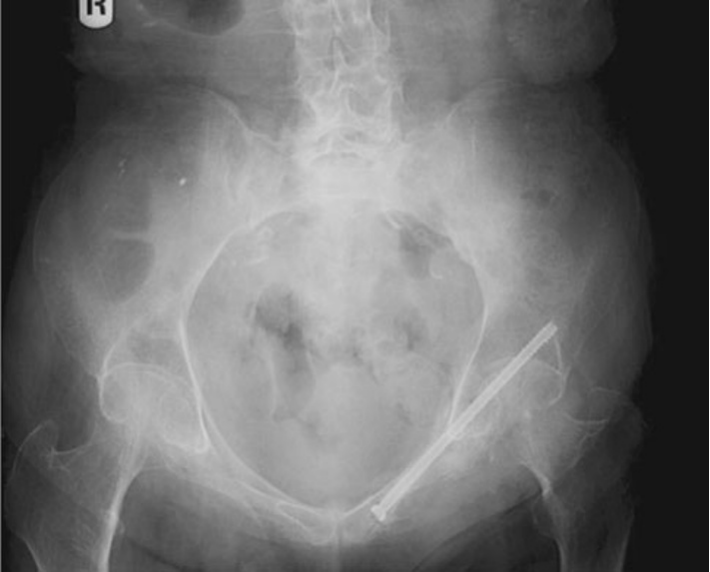
Inlet view at follow-up

**Fig. 6:**
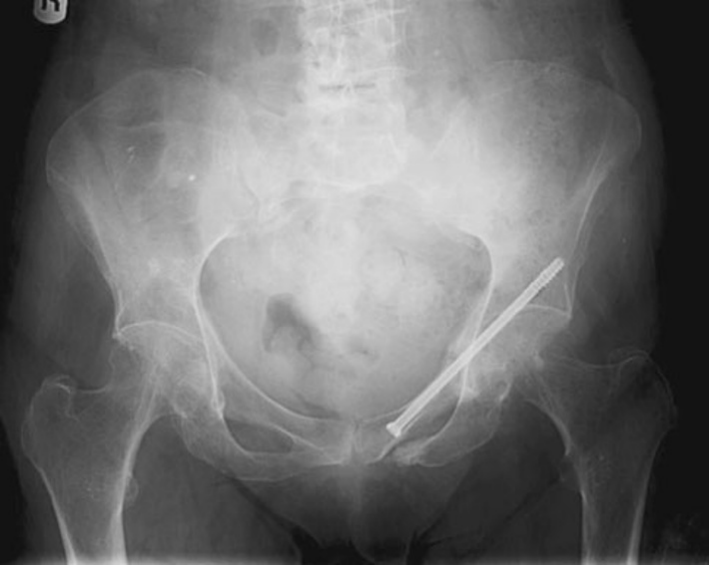
Outlet view at follow-up

**Fig. 7:**
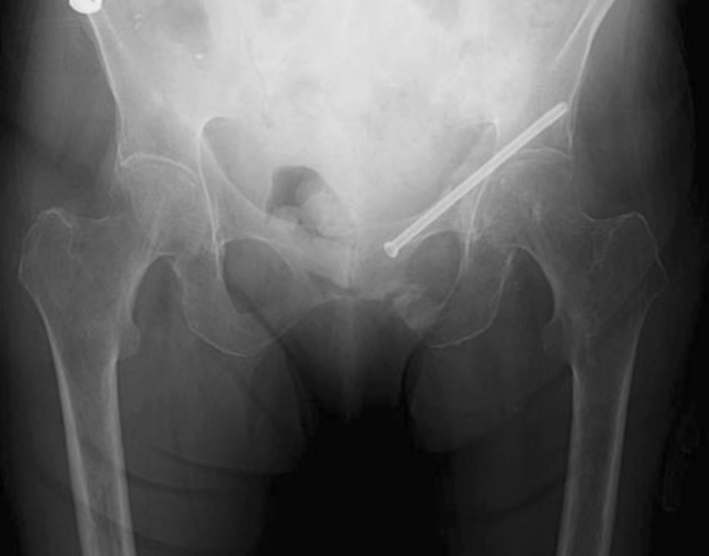
Standard AP pelvis view at follow-up

The operative technique was described by Routt et al. [11] and a similar technique by Mosheiff et al. [10]. Biplanar fluoroscopy was used with inlet and obturator oblique views. The inlet view identifies the anterior pelvic ring anatomy. The obturator oblique view is obtained by combining the outlet tilt with 20 degrees of lateral C-arm rotation. This combination image demonstrates the safe zone for screw placement within the pubic ramus, including the area superior to the acetabulum. Intravenous cephalosporin prophylaxis (Kefzol ^®^ 2,000 mg, Lilly^®^, Houten, The Netherlands) was given once at the start of anaesthesia. A 2.4-mm K-wire was then inserted in a retrograde direction within the distal medial fragment of the superior ramus pubis under biplanar inlet and obturator oblique fluoroscopic control (Figs. 1, 2, 3 and 4) The K-wire is moved forward in a straight line cephalad to the hip joint. The appropriate length 6.5 mm partially threaded cannulated cancellous screw (Stryker, Waardenburg, The Netherlands) was then inserted. The patients received Fraxiparine^®^ 0.3 ml (9,500 IE/ml, GlaxoSmithKline, Zeist, The Netherlands) during their hospital stay with weightbearing allowed from the first day post-operatively, combined with muscle-strengthening exercises under the supervision of a physiotherapist. Plain inlet and outlet pelvic radiographics were obtained on the first day post-operatively and during the follow-up period (Figs. 5, 6 and 7). The patients were discharged when pain had decreased and weightbearing was possible.

## Results

Eight retrograde superior pubic ramus screws were inserted percutaneously in six patients, with two patients having had bilateral fractures. The median time between injury and operation was 5 days (range 3–42). The median post-operative hospital stay was 6 days (range 3–18). No screw violated the acetabulum. Post-operatively, one patient had pneumonia, two had confusional states, and one patient had a urinary tract infection. No wound infections or osteomyelitis occurred. After surgical intervention, three patients were pain free and three patients had substantial pain reduction. Four patients had regained their pre-trauma mobility, and two patients showed an improvement. Four patients were discharged to their original residence, and two patients were discharged to a rehabilitation unit. No screw breakage or loosening was observed. At 1-year follow-up, one patient had died, four were as mobile as before the fracture and without pain, and one patient was less mobile due to severe hip osteoarthritis.

## Discussion

Pubic ramus fractures interfere with mobility due to pain. Elderly patients with isolated pubic ramus fractures utilize substantial healthcare resources owing to the length of hospital and rehabilitation unit stay. As reported by Hill et al., most patients with a pubic ramus fracture do not change their residential status after discharge from the hospital, but after a mean hospital stay of 9–14 days, 44 % of these patients need further rehabilitation in a geriatric orthopaedic unit; the mean time spent in this unit was 41 days [13]. There was a significant decline in mobility with 53 % of the patients not reaching their pre-injury levels by discharge; 40 % of surviving patients had not regained their previous level of mobility after 5-year follow-up [6].

Although a fracture of the pubic ramus is the most common fracture of the pelvis, little has been reported regarding the treatment of these fractures. Most studies are concerned with pelvic fractures and do not specifically address (isolated) fractures of the pubic ramus. Pubic ramus fractures typically heal uneventfully. To our knowledge, this is the first report of cannulated screw fixation for subacute pubic ramus fracture(s) in the elderly. Although small and retrospective, this series suggests percutaneous cannulated screw fixation can be a good and safe method to decrease pain and improve rehabilitation. The technique used in this study was described for pelvic fractures associated with posterior pelvic injury [11, 13] and in non- or malunions of the pubic ramus [1, 2]. Non-unions of the pubic ramus are rare and may be asymptomatic, but when they are refractory to conservative treatment, percutaneously inserted retrograde superior pubic ramus screws are an alternative with good outcome [1, 2, 4, 9, 14]. The successful treatment of an 83-year-old woman with persisting pain caused by a superior pubic ramus pseudo-arthrosis led to the idea of acute treatment of these fractures.

Biomechanical tests in unstable anterior pelvic ring fractures showed that the retrograde intramedullary screw is equivalent to standard plating techniques [13]. The technique using the retrograde intramedullary screw avoids extensive surgical exposure and avoids complications of anterior pelvic plating [3, 5]. Accurate screw insertion requires excellent intra-operative fluoroscopy and an understanding of pelvic ring anatomy [2]. Possible difficulties associated with the screw placement include the inability to insert the screw due to anatomical variations, extramedullary misdirection of the screw and post-operative disengagement [11]. In our study, we observed no operation-related complications.

Mosheiff et al. [10] described a technique that enables the use of a relatively thick retrograde intramedullary screw in difficult anatomical variations such as narrow and curved pubic rami. As long as the retrograde medullary screw is in the medial fragment, the screw can be manoeuvred to improve reduction. Akagi et al. [1] described a modification of the technique in which the screw orientation was altered, so that it engaged the cancellous bone in the inferior part of the anterior column and the antero-inferior cortex of the fossa acetabuli. This modification is an option when the original technique is impossible.

This study suggests that retrograde screw fixation of fractures of pubic ramus in elderly patients is a valid treatment option. Incorporation of this option in the treatment algorithms is a potential refinement of the contemporary treatment algorithms that has to be further evaluated. The use of well-defined and validated functional outcome measures, including quality of life measures and VAS pain scores, are preferable in the evaluation of this treatment modality as is the cost-effectiveness of the technique.
